# Supplementary material for: Batch alignment of single-cell transcriptomics data using deep metric learning
Source: Nat Commun. 2023 Feb 21;14:960. doi: 10.1038/s41467-023-36635-5 (PMC9944958; doi:10.1038/s41467-023-36635-5)
Supplement: Supplementary file 1 — Supplementary Information [file 41467_2023_36635_MOESM1_ESM.pdf]

Supplemental Information

Batch alignment of single-cell transcriptomics data using deep metric learning

Xiaokang Yu[#], Xinyi Xu[#], Jingxiao Zhang[*], Xiangjie Li[*]

***Correspondence:**

Xiangjie Li, Ph.D, xiangjieli@cpl.ac.cn

Jingxiao Zhang, Ph.D, zhjxiao@ruc.edu.cn

[#]These authors contributed equally: Xiaokang Yu and Xinyi Xu**.**

Supplemental Table 1: Methods compared with scDML

Method	version	URL	Reference
FastMNN	1.10.0	https://bioconductor.org/packages/release/bioc/html/batchelor.html	[1]
Harmony	0.1.0	https://github.com/immunogenomics/harmony	[2]
Seurat 3	4.1.1	https://satijalab.org/seurat/	[3]
Liger	1.0.0	https://github.com/welch-lab/liger	[4]
INSCT	0.0.2	https://github.com/lkmklsmn/insct	[5]
BERMUDA*	-----	https://github.com/txWang/BERMUDA	[6]
BBKNN	1.5.1	https://github.com/Teichlab/bbknn	[7]
Scanorama	1.7.2	https://github.com/brianhie/scanorama	[8]
scVI	0.8.1	https://github.com/scverse/scvi-tools	[9]
carDEC	1.0.3	https://github.com/jlakkis/CarDEC	[10]

*BERMUDA algorithm has no version number.

Supplemental Table 2: Hyperparameters for scDML

**Hyperparameter**	**Default**	**Brief Description**
n_hvg_var	1000	the number of genes to be retained as HVGs
scale_value	10.0	the maximum value set in the step of z-score normalization
pca_dim	100	the number of components chosen in PCA dimension reduction
cluster_method	Louvain	clustering algorithm to initialize the cluster label
resolution	3.0	default resolution chosen in louvain algorithm
K_in	5	the number of neighbors chosen in calculating KNN pair intra batch
K_in_metric	cosine	the distance function chosen in calculating KNN pair intra batch
K_bw	10	the number of neighbors chosen in calculating MNN pair inter batch
K_out_metric	cosine	the distance function chosen in calculating MNN pair inter batch
expect_num_cluster	None	The number of clusters after merging. It can be specified by the user, otherwise it will be calculated by scDML with default settings
dims	[256,32]	list of layer sizes for the embedding net, excluding the input layer which is inferred from the data
epochs	50	the maximum number of iterations for training the scDML model, described in the Step 3 of the workflow
batch_size	64	batch size for minibatch gradient descent
distance function	Euclidean	the distance function chosen in calculating triplet loss
optimization method	Adam	the optimization method chosen for gradient descent
learning rate	0.01	learning rate for minibatch gradient descent
margin	0.2	the hypermeter of triplet loss which indicates the margin between different clusters
triplet type	hard	the triplet type chosen for training of deep metric model

Supplemental Table 3: Hyperparameters used in scDML for all datasets

Dataset	K_in	K_out	N_hvg	Cluster_ method	Resolu tion
Simulation1	5	10	1000	louvain	3.0
Simulation2	5	10	1000	louvain	3.0
Mammary epithelial (bct)	5	10	1000	louvain	3.0
Mammary epithelial with delete celltype (bct_del)	5	10	1000	louvain	3.0
Mouse retina	5	10	1000	louvain	3.0
Pancreas	5	10	1000	louvain	3.0
Macaque retina	5	10	1000	louvain	3.0
Human_mouse_ lung	5	10	1000	louvain	3.0
FullMouseBrain	5	20	1000	leiden	6.0
Failing humn heart (45 batches)	5	20	1000	leiden	6.0
Healthy human heart (140 batches)	5	20	2000	leiden	6.0

Supplemental Table 4: The number of clusters estimated by Algorithm 3 for all datatets

dataset	The number of truecelltype	The number of clusters estimated by Algorithm3 (topK=5)
bct	3	[3,4,7,12 ,15]
bct_del	3	[2,3,6,14,12]
Simulation1	4	[6,4,8,10,14]
Simulation2	7	[2,11,7,4,14]
Pancreas	13	[7,6,4,16,27]
Macaque retina	12	[8,12,18,6,5]
Lung_two_species	17	[5 10 14 17 20]
Mouse retina	14	[3,1,6,11,2]
Failing human heart (45 batches)	14	[7,11,13,20,23]
Healthy human heart (140 batches)	13	[8,10,1,15,18]
FullMouseBrain	14	[8,10,14,20,27]

Supplementary Note

Step1: preprocessing

There are five important tasks to be completed in preprocessing: filtering low-quality cells and genes, cell normalization, log normalization, detecting highly variable genes (HVGs), and z-score normalization with truncated values. All the above steps are implemented in the python module *scanpy* [11] with version 1.7.2.

Let **X** be an $n \times p$ matrix of scRNA-seq data, with n cells, p genes and M batches, and let x_{ij} be the expression value of gene j in cell i. In the filtering step, we first remove any cells expressing less than 10 genes and remove any genes expressed in less than 3 of the remaining cells. In cell normalization, we divide counts for each cell by the total counts over all genes and then multiply a constant (10000 by default) using the function *sc.pp.normalize_total*, so that each cell has the same total UMI counts after cell normalization. The normalized count y_{ij} can be denoted by

$$y_{ij} = x_{ij} \cdot \frac{10000}{\sum_{j=1}^{p} x_{ij}} (i = 1 \cdots n, j = 1 \cdots p). \quad (1)$$

To reduce the influence of outlier, we then conduct log transformation for y_{ij} (using *sc.pp.log1p*). In detail, we simply add a pseudo count and then take the natural logarithm elementwise,

$$y_{ij} = log\,(y_{ij} + 1). \quad (2)$$

Then the log-normalized counts are used to determine which genes are highly variable. Specifically, we call the function *sc.pp.highly_variable_genes* with parameter *n_top_genes* defining the number of HVGs to keep. In our scDML method, we set *n_top_genes* = 1000.

Lastly, we conduct z-score standardization for updated y_{ij}. Rather than doing a simple z-score standardization across all cells, we conduct z-score normalization within each batch and truncate the expression with a maximum value *t*. More precisely, suppose we have batches b_m for m = 1, 2, …, M. Let B_m be the set of cells sequenced in batch b_m. Then for each gene j, we compute batch-specific mean $\mu_{m,j}$ and variance $\sigma_{m,j}^2$ for each batch m as follows,

$$\mu_{m,j} = \frac{\sum_{i \in B_m} y_{ij}}{\sum_{i \in B_m} 1}, \quad (3)$$

$$\sigma_{m,j}^2 = \frac{\sum_{i \in B_m} (y_{ij} - \mu_{m,j})^2}{(\sum_{i \in B_m} 1) - 1}. \quad (4)$$

Then, we conduct z-score transformation for each y_{ij} using the mean and variance of the batch corresponding to cell i. Specifically, let m_i be defined such that $i \in B_{m_i}$.

Then the batch-specific z-score normalization is performed as follows:

$$y_{ij} = \min\left(\frac{y_{ij}-\mu_{m_i,j}}{\sqrt{\sigma^2_{m_i,j}}}, t\right). \tag{5}$$

In our scDML algorithm, we set $t = 10.0$ by default.

Step2: Initializing clusters in PCA embedding space

Let $\mathbf{Y}_{HVG}$ be the $n \times p_{HVG}$ matrix of normalized expression from **Step 1**, including only the p_{HVG} highly variable genes. Let $y_{i,HVG}$ be the expression value of HVGs in cell i, i.e., the ith row of $\mathbf{Y}_{\mathrm{HVG}}$. To get a suitable initial clustering result for scDML, we firstly conduct PCA (Principal Component Analysis) on $\mathbf{Y}_{HVG}$ to get a low-dimensional embedding X_{emb}. Unless otherwise stated, we set the number of principal components n_{pca} to be 100 without losing too much information. It is easily implemented in *scanpy* package with the function *sc.tl.pca* (*n_components*=100). Then scDML applies the Louvain [12] method, a graph-based clustering method that has been shown to outperform other clustering methods, on the reduced PCA embedding space to get an initialized clustering result. This procedure can be implemented by the function *sc.tl.louvain* in *scanpy* package, higher resolution means finding more and smaller clusters. However, the number of true cell types is usually unknown in real data, so how to find the right resolution for the Louvain algorithm is a challenge. scDML set a relatively large resolution (3.0 by default) in the Louvain algorithm, which may help to find more subtle cell types in datasets. Suppose we get C clusters with the Louvain algorithm and m_j is the number of cells in the jth cluster, for $j = 1,2 \cdots C$. In most situations, this resolution results in more clusters whose number is larger than the true number of cell types. To remove batch effect in the dataset, it is natural to merge similar clusters intra and inter batches.

Step3: Finding NN pairs in PCA embedding space

To merge the initialized clusters obtained from the Louvain algorithm in **Step2**, we need to compute the similarity between the clusters. Similar to Conos algorithm[13], scDML firstly builds a joint graph between all clusters by finding NN (Nearest Neighbor) pairs intra batch and inter batches.

3.1 Finding KNN pairs intra batch

Let $X_{emb} = (\mathrm{X}^1, \dots, \mathrm{X}^M)^T$ be a $n \times n_{pca}$ matrix of scRNA-seq data in PCA embedding space, where $\mathrm{X}^k (k = 1,2 \cdots M)$ is $n_k \times n_{pca}$ submatrix of cells in the kth batch. Let x_i^k be the vector of cell i of batch k in PCA embedding space, that is, the ith row of X^k. Denote S_k as the set of all KNN pairs in batch k, cell i and cell j form a KNN pair if and only if

$$(i,j) \in S_k \; and \; (j,i) \in S_k \Leftrightarrow \begin{cases} i,j \in B_k \\ i \in \mathrm{KNN}(x_j^k) \; or \; j \in \mathrm{KNN}(x_i^k) \end{cases} \tag{6}$$

where the tuples (i,j) and (j,i) are both KNN pairs, B_k is the set of cells belonging to the batch k. For the clarity of the following description, we treat (i,j) and (j,i) as different NN pairs. $\mathrm{KNN}(x_j^k)$ represents the set of k-nearest neighbors of cell i in batch k. To find KNNs intra batches, we set the number of neighbors K_{in}=5 and uses cosine distance for scDML.

3.2 Finding MNN pairs inter batches

Many studies have proved that MNN (mutual nearest neighbor) based methods can effectively remove batch effect in scRNA-seq data such as MNN[14],BEER[15], BBKNN[7], Scanorama[8], and INSCT[5].

So here we also use the MNN pairs to construct the similarity of clusters between different batches. To correspond with the definition of KNN pairs intra batch, let $S_{a,b}$ be the set of MNN pairs between batch a and batch b, then cell i and cell j form an MNN pair if and only if:

$$(i,j) \in S_{a,b} \; and \; (j,i) \in S_{a,b} \Leftrightarrow \begin{cases} i \in B_a, j \in B_b \\ i \in \mathrm{MNN}(x_j^b, X^a) \; and \; j \in \mathrm{MNN}(x_i^a, X^b) \end{cases}, \quad (7)$$

where the tuples (i,j) and (j,i) are both MNN pairs, $\mathrm{MNN}(x_i^a, X^b)$ represents the sets of cells in batch b which are nearest to cell i in batch a, and $\mathrm{MNN}(x_j^b, X^a)$ represents the sets of cells in batch a which are nearest to cell j in batch b. scDML sets the number of neighbors K_{bw}=10 and uses cosine distance to calculate MNN pairs.

Step4: Calculate similarity of clusters

Let S_{in} represents all KNN pairs intra batches, S_{bw} represents all MNN pair inter batches and S represents all *NN* pairs in the dataset.

$$S_{in} = S_1 \cup S_2 \cup \cdots \cup S_M, \quad (8)$$

$$S_{bw} = \cup_{j=1}^{M} \cup_{j=i+1}^{M} S_{ij}, \quad (9)$$

$$S = S_{in} \cup S_{bw}, \quad (10)$$

Let $N = |S|$ denote the total number of *NN* pairs in set *S*. Based on the clustering results in Step2 and all *NN* pairs obtained in Step 3, we calculate the number of NN pairs between pairwise clusters, and define a symmetric matrix A as

$$\mathrm{A} = \begin{bmatrix} a_{1,1} & a_{1,2} & \cdots & a_{1,C} \\ a_{2,1} & a_{2,2} & \cdots & a_{2,C} \\ \vdots & \vdots & \ddots & \vdots \\ a_{C,1} & a_{C,2} & \cdots & a_{C,C} \end{bmatrix}, \quad (11)$$

where $a_{i,j}$ represents the number of NN pairs between cluster i and cluster j. Obviously, the smaller the cluster size is, the less the number of NN pairs will be found. So, we should take the cluster size (the number of cells in each cluster) into

consideration when using A to represent the similarity (or connectivity) between clusters. To achieve the above goals, scDML adopts a simple but intuitive method to calculate the similarity matrix between clusters,

$$S = \begin{bmatrix} s_{1,1} & s_{1,2} & \cdots & s_{1,C} \\ s_{2,1} & s_{2,2} & \cdots & s_{2,C} \\ \vdots & \vdots & \ddots & \vdots \\ s_{C,1} & s_{C,2} & \cdots & s_{C,C} \end{bmatrix}, \ s_{i,j} = \frac{a_{i,j}}{\min(m_i, m_j)}, i, j = 1, \ldots, C, \quad (12)$$

where m_i denotes the number of cells in cluster i, and the matrix S is still symmetric. K_{in} and K_{bw} are parameters to calculate NN pairs defined in **Step3**.

In particular, scDML applies a new merging rule different from BERMUDA, which can generate a more stable result. scDML utilize the idea of hierarchical clustering to merge clusters one by one. For the detailed merging procedure please refer to Algorithm 1.

Algorithm 1: Merging rule for Nearest Neighbor pairs

Input: K(Number of expected clusters)
S(Similarity matirx)
A(Nearest Neighbor pair matrix)
C(Number of initial clusters by Louvain)
$\boldsymbol{m}$(Cluster size for each cluster)

Output: P(set of merging cluster)

$P \leftarrow \emptyset, n \leftarrow C, S' \leftarrow S$
for i=*1 to* n **do**
  $P \leftarrow P \cup \{i\}$
end
while n>K **do**
  Step1: find most similar cluster in similarity matrix
  minval=-∞
  count = 1
  for i =*1 to* n **do**
    for j=i+*1 to* n **do**
      if $S'_{i,j} \geq minval$ **then**
        $minvalue \leftarrow S'_{i,j}$
        $pos1 = i, pos2 = j$
    end
  end
  Step2: merge cluster i and cluster j to custer k
  delete $pos1, pos2$ rows of similarity S'
  delete $pos1, pos2$ columns of similarity S'
  $P \leftarrow P \setminus \{pos1\}$
  $P \leftarrow P \setminus \{pos2\}$
  $P \leftarrow P \cup \{n + count\}$
  $m_{n+count} \leftarrow m_{pos1} + m_{pos2}$
  for $j = 1$ *to* $n - 2$ **do**
    $S'_{n-1,j} \leftarrow \frac{A_{pos1,j}+A_{pos2,j}}{min(m_{n+count},m_j)}$
  end
  for $i = 1$ *to* $n - 2$ **do**
    $S'_{i,n-1} \leftarrow \frac{A_{i,pos1}+A_{i,pos2}}{min(m_{n+count},m_i)}$
  end
  $n \leftarrow n - 1$
  $count \leftarrow count + 1$
end

After the above merging procedure, we finally obtain a set P whose element can be viewed as an edge in an undirected graph G, in which the node of G is the clustered index $\{1, \ldots, C\}$. Then we can find all connected components in graph G to reassign

the label of the cluster, and it can be easily implemented in the python module *networkx* with the function *nx.connected_components*(*G*). Suppose that we have obtained *K* connected components in graph *G*. We represent each connected component as G_i, $i = 1, \ldots, K$, where each G_i is the subset of $\{1, \ldots, C\}$ and they are disjoint. That is to say,

$$\cup_{i=1}^{K} G_i = \{1, \ldots, C\},\ G_i \cap G_j = \emptyset, \forall i \neq j, i = 1, \cdots K, j = 1, \cdots K. \quad (13)$$

K can be set as the final number of expected clusters. In other words, all clusters belonging to a same connected component should be considered as one cluster so that we can merge the initialized clusters to the updated clusters, taking the batch effect into consideration.

When there is no prior knowledge about the number of clusters to be finally defined, scDML provides a rough estimation about the parameter (K) based on spectral clustering and eigen decomposition. Please refer to in Algorithm3 for details.

Algorithm 3: Estimating the number of clusters using similarity matrix between initial clusters for scDML

Input: S:similarity matrix S; $topK$: the number of candidate clusters

Output: The suggested number of clusters eigengap heuristic.

Step 1: Normalize similarity matrix to $[0, 1]$

$$S_{norm} = \frac{S}{max(S)}$$

Step 2: enhance the cluster structure

$$S'_{norm} = S_{norm} + S^2_{norm}$$

Step 3: calulate Laplacian graph matrix from S'_{norm}

$$A = D^{-\frac{1}{2}} S'_{norm} D^{-\frac{1}{2}}$$

Step 4: calculate the eigenvalue of A

Step 5: sort the eigenvalues in descending order and compute the $topK$ **larger gap between eigenvalues.**

The indexes corresponding to the $topK$ largest gap were regarded as the optimal number of clusters.

Step5: Deep Metric Learning to remove batch effect

As we know, MNN pairs can help to remove batch effect to some degree. Therefore, we tend to use the above MNN-guided information to build better low-dimensional embeddings. In addition, metric learning is an approach based on distance metric directly, which aims at automatically constructing task-specific from (weakly)

supervised data[16,17]. Although scDML has reassigned the cluster label of the dataset, raw data has not been corrected for batch effect actually. Here we use the deep metric learning (DML) method with triplet loss to capture more accurate low-dimensional representation. Broadly speaking, our goal is to learn a distance metric that pulls points with the same label close together while pushing away points with different labels, meanwhile considering the influence of batch effect.

5.1 Triplet Definition

In step **4**, we have obtained the cluster label for each cell. To make full use of the cluster information, we construct triples (anchor, positive, negative) according to the following guidelines. Given a cell a **(anchor point)**, we randomly choose a cell p as a positive point from cells whose cluster label is the same as a and randomly select a cell n as a negative point from cells whose cluster label is different from a. The tuple (a, p, n) can be regarded as a triplet. Any cell in the dataset can be used as an anchor.

5.2 Triplet Loss

Here, the triplet loss function is defined as follows:

$$L(a, p, n) = \max(d(a, p) - d(a, n) + m, 0), \quad (14)$$

where d is the distance metric and we adopt Euclidean distance. m is a margin between similar and dissimilar pairs, by default m=0.2. The triplet loss is optimized by minimizing the distance between anchor–positive pairs and maximizing the distance between anchor–negative pairs. Based on the definition of the triplet loss, there are three possible categories of triplets:

1) **Easy triplets**: triplets which have a loss of 0, that is, $d(a, p) + m < d(a, n)$;
2) **Hard triplets**: triplets where the negative point is closer to the anchor point than the positive point, i.e., $d(a, n) < d(a, p)$;
3) **Semi-hard triplets**: triplets where the negative is not closer to the anchor than the positive, but the loss is still positive. i.e. $d(a, p) < d(a, n) < d(a, p) + m$

As can be seen from above, easy triplets do not affect the optimization of triplet loss. Semi-hard triplets can be used for the optimization, but it will find too many triplets, which will cost much training time and memory for real datasets. Considering the time and memory consumption, we select hard triplets found in embedding space to train the scDML model.

5.3 The structure of scDML model and training

Unless otherwise specified, scDML adopts a simple fully connected three-layers network. The number of nodes of the input layer, the hidden layer and the embedding layer is 1000 (or 2000), 256 and 32 respectively. The input dimension of embedding layer depends on the number of highly variable genes used in the preprocessing step. We used ReLU as the activation function, and each layer is fully connected. According to the definition of triplets, the anchors are independent, and thus we can optimize the triplet loss by a mini-batch strategy. Algorithm 2 illustrates the detailed

training procedure, in which $f(\cdot)$ represents the nonlinear function. The implementation of scDML is based on pytorch framework, which makes full use of a scalable package named *pytorch_metric_learning*.

Algorithm 2: Mini-batch training of scDML

Input: Batch size B, training epochs n_{epoch}, margin m, distance function $d(.)$, normalized data matrix X with HVGs, and ressigned cluster label for each cell y.

Output: low-dimensional embedding $X_{\text{corrected}}$ whose batch effect is removed

Initialize the weights of scDML network.
for $i = 1$ **to** n_{epoch}: **do**
  for $b = 1$ **to** $[n/B]$ **do**
    Step 1: Construct batch samples
    Subsample from X to get the batch samples $X_{sub} = \{x_1, \cdots, x_B\}$
    Step 2: Compute embedding
    Calculate embedding for $f(x_1), \cdots, f(x_B)$
    Step 3: Find all hard triplets
    Compute all hard triplets(a, p, n) satisfying

$$d(f(x_a), f(x_p)) < d(f(x_a), f(x_n)), x_a, x_p, x_n \in X_{sub}$$

    Denote S_b be the set of hard triplets
    Step 4: Minimize triplet loss
    Minimize triplet loss $L(f(x_a), f(x_p), f(x_n), m)$ using S_b

$$L = \sum_{(x_a, x_p, x_n) \in S_b} max(d(f(x_a), f(x_p)) - d(f(x_a), f(x_n)) + m, 0)$$

  end
end
Obtain the final embedding $X_{\text{corrected}}$

Supplementary Figures

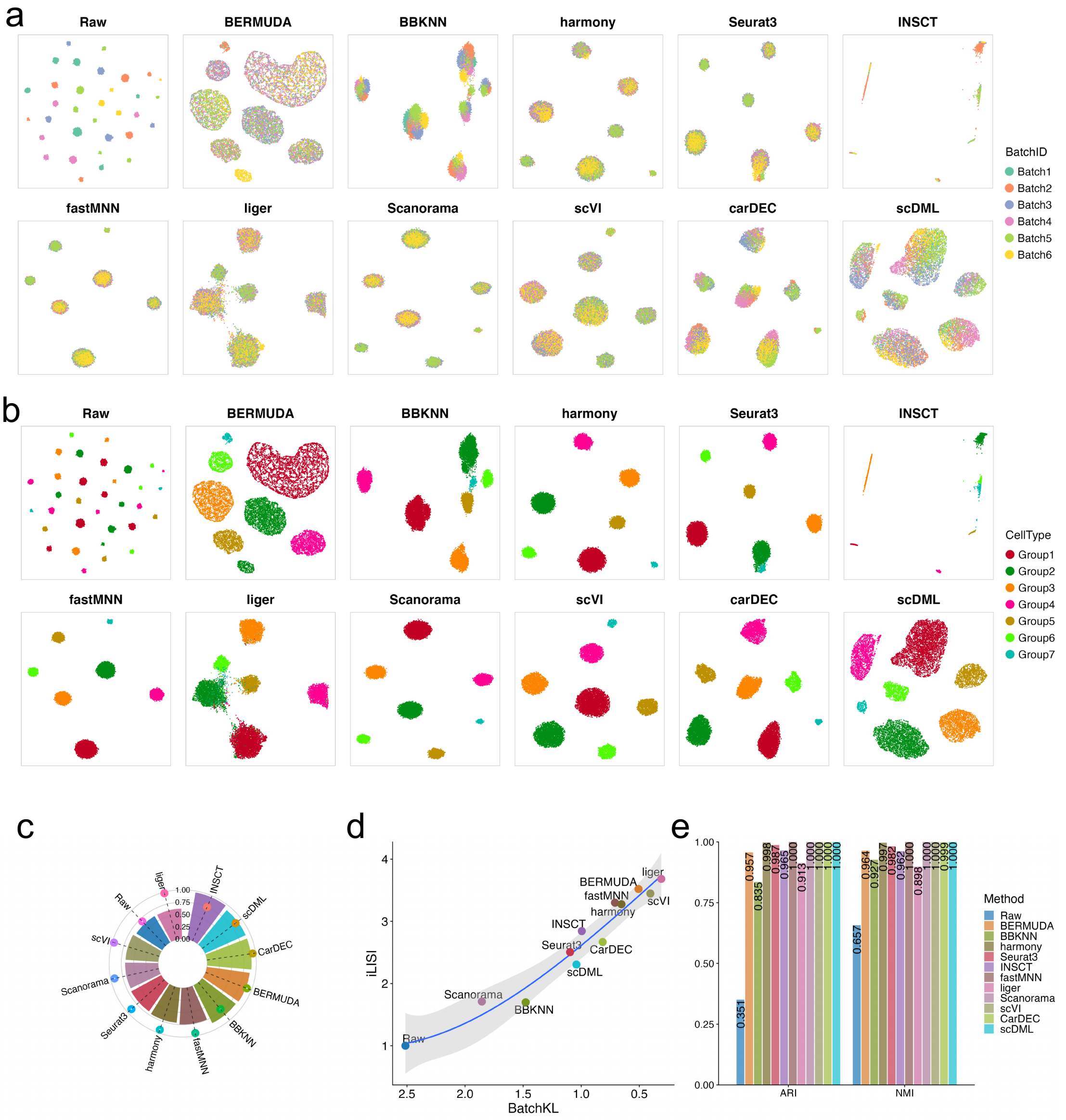

Figure S1. **scDML removes batch effects and keeps the biological difference in the simple simulated scRNA-seq data.**

(a) UMAP embedding computed from compared methods, in which the points are colored by batch.

(b) UMAP embedding computed from compared methods, in which the points are colored by cell type.

(c) Bar plot shows the value of ASW_celltype and ASW_batch, in which the bar height denotes the value of ASW_celltype and the point height denotes the value of ASW_batch. Higher ASW_celltype and lower ASW_batch means better performance.

(d) Scatter plot shows the value of BatchKL (x-axis) and iLISI (y-axis). Point closer to the upper right means better performance. The error band means confidence

interval of 0.95 level around smooth using B-spline smoothing function with degree equal to 3.
(e) Bar plot shows the value of ARI and NMI for different methods. Higher bar means better performance.

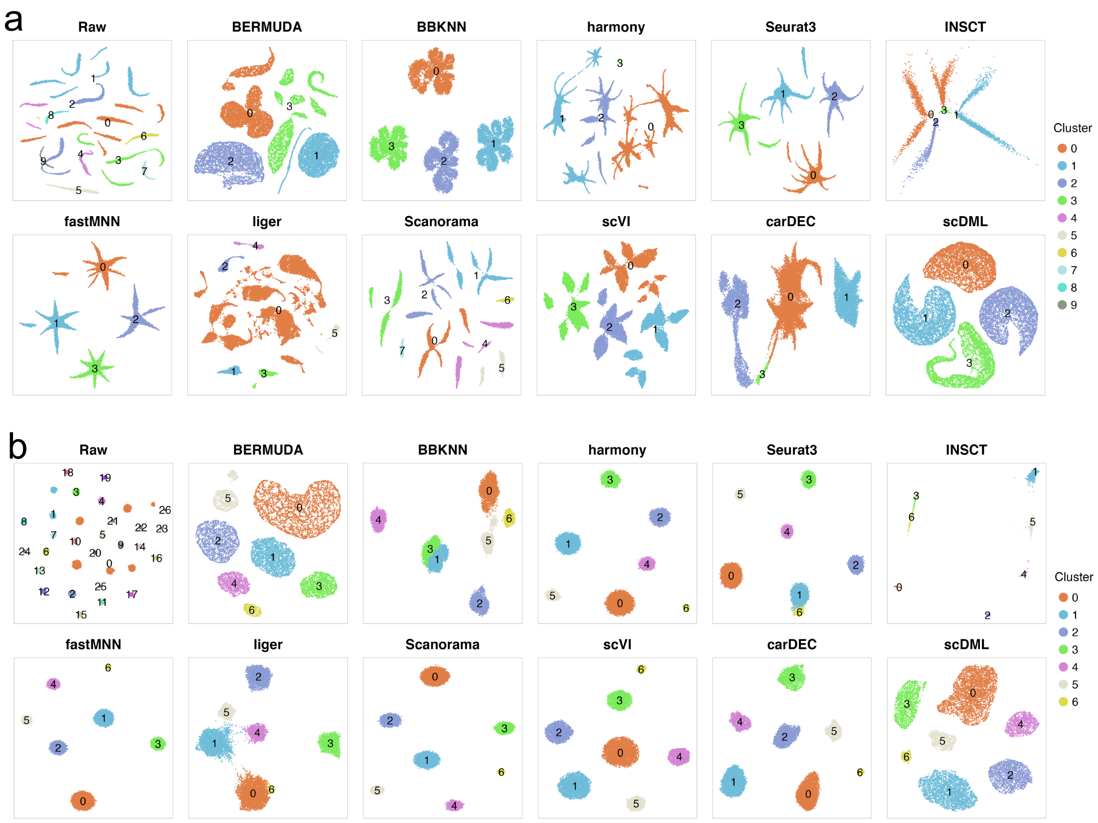

Figure S2. UMAP plot shows the clustering result for different methods.
(a) UMAP plot shows the cluster result for different methods in the complex simulated dataset, in which the points are colored by cluster label.
(b) UMAP plot shows the cluster result for different methods in the simple simulated dataset, in which the points are colored by cluster label.

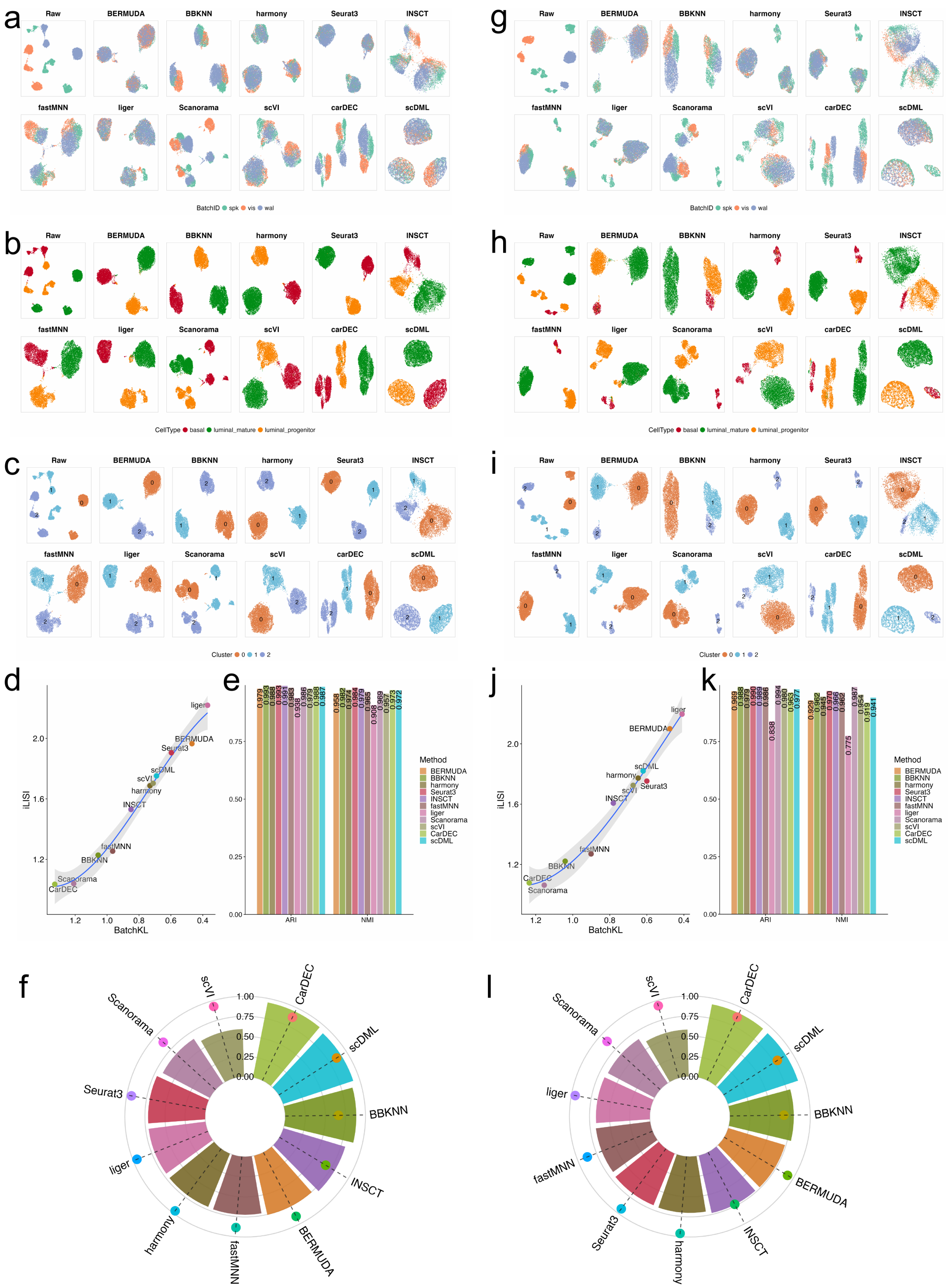

Figure S3. scDML removes batch effects and keeps the biological difference in the mammary epithelial dataset.

(a)-(f) for the real mammary epithelial dataset:

UMAP embedding computed from different methods, in which the points colored by batch (a), by cell type (b) and by cluster label (c).

(d) Scatter plot shows the value of BatchKL (x-axis) and iLISI (y-axis). Point closer to the upper right means better performance. The error band means confidence

interval of 0.95 level around smooth using B-spline smoothing function with degree equal to 3.

(e) Bar plot shows the value of ARI and NMI for different methods. Higher bar means better performance.

(f) Bar plot shows the value of ASW_celltype and ASW_batch, in which the bar height denotes the value of ASW_celltype and the point height denotes the value of ASW_batch. Higher ASW_celltype and lower ASW_batch means better performance.

(g)-(l) for the modified mammary epithelial dataset where cell type *basal* only exists in batch *spk*:

The implication of (g)-(l) is correspondingly the same as (a)-(f).

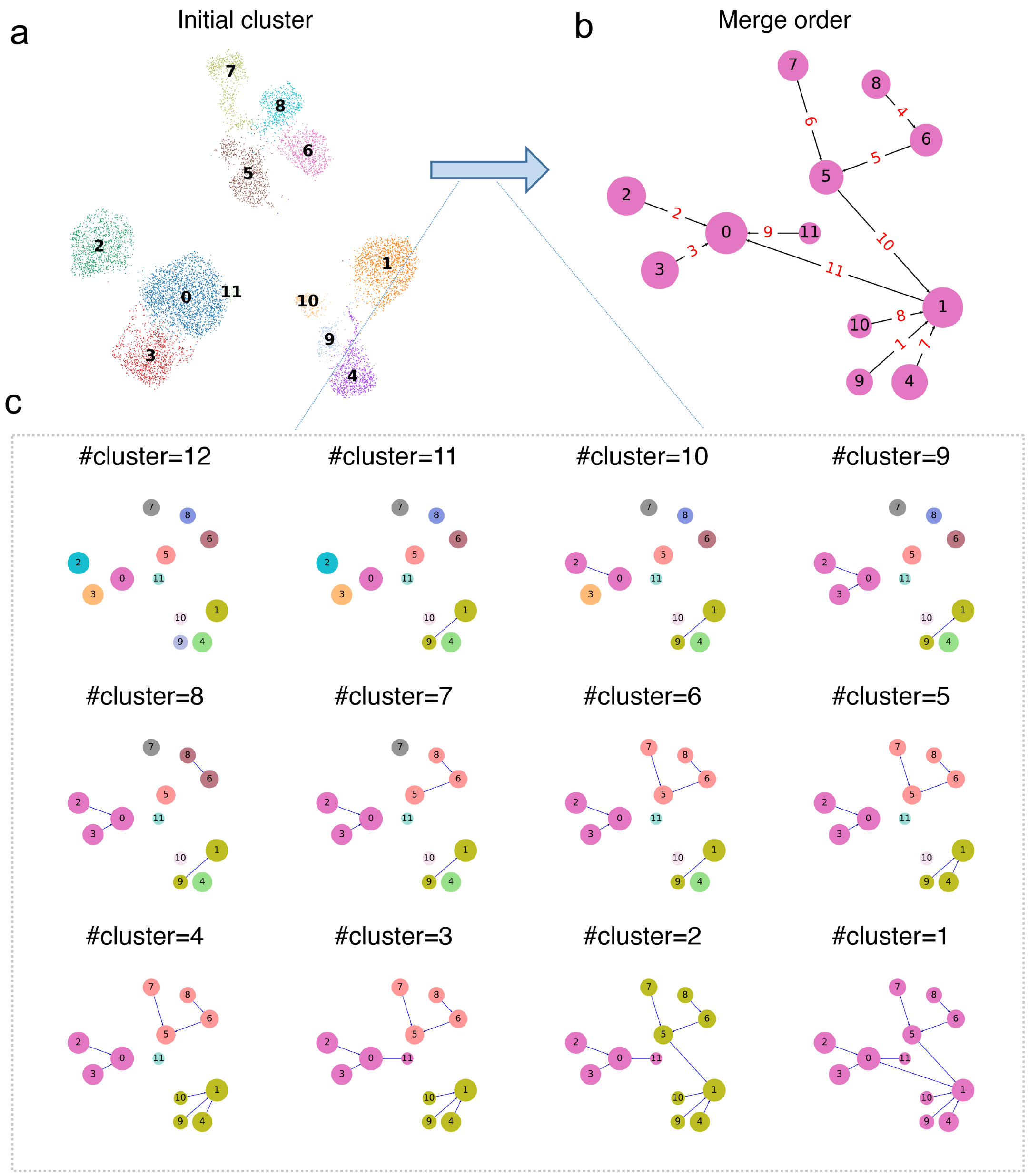

Figure S4. The schematic diagram of the merge process for scDML in the mammary epithelial dataset.

(a) UMAP embedding shows the initial clusters.

(b) The overall merge orders.

(c) The detail merge order in each step.

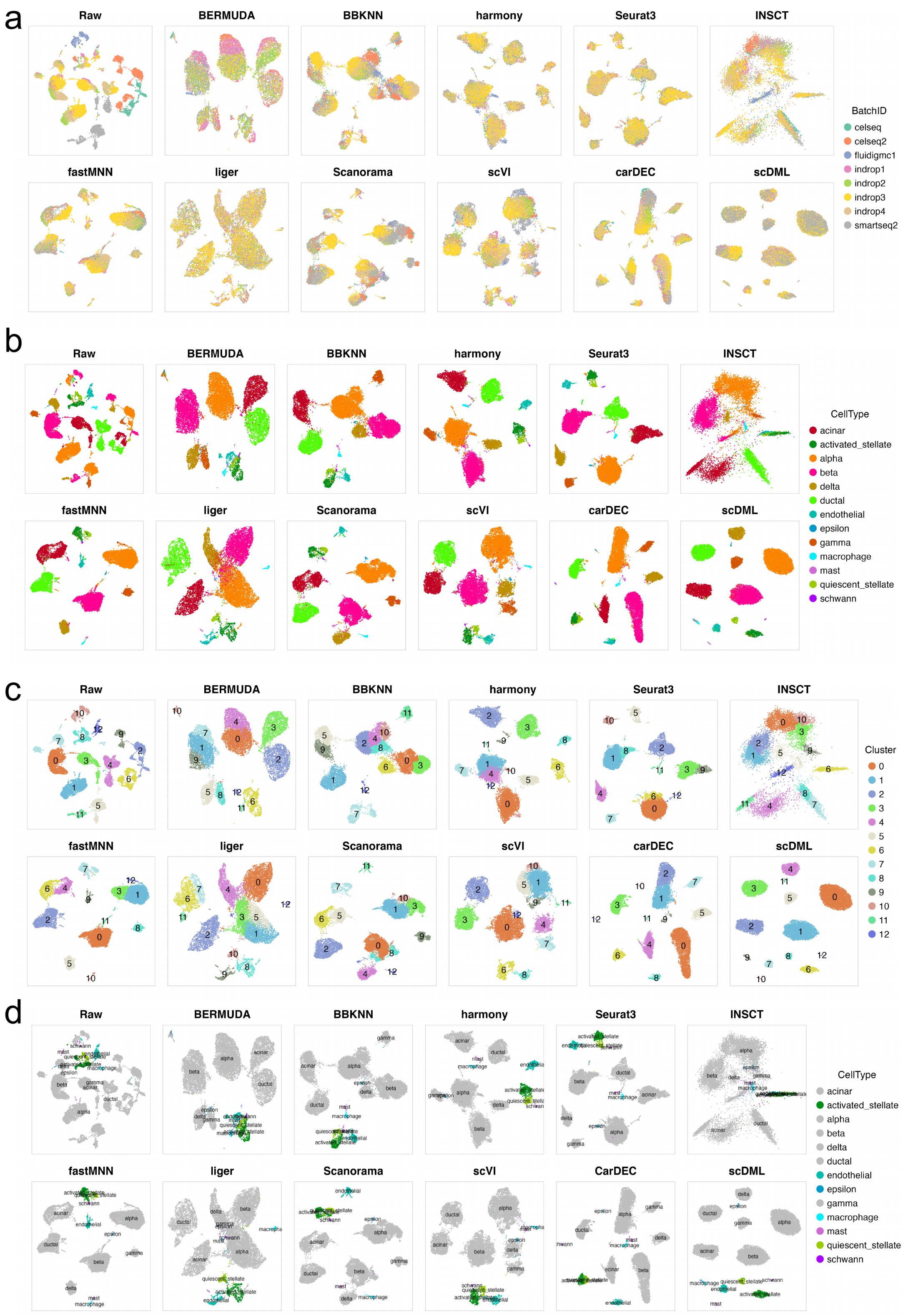

Figure S5. scDML accurately perserves the rare cell type and removes batch effect for the pancreas dataset.

UMAP embedding from different methods, and the points are colored by batch (a), by cell type (b) and by cluster label (c).

(d) UMAP embedding computed from different methods, and the highlighted cell types are some rare cell types.

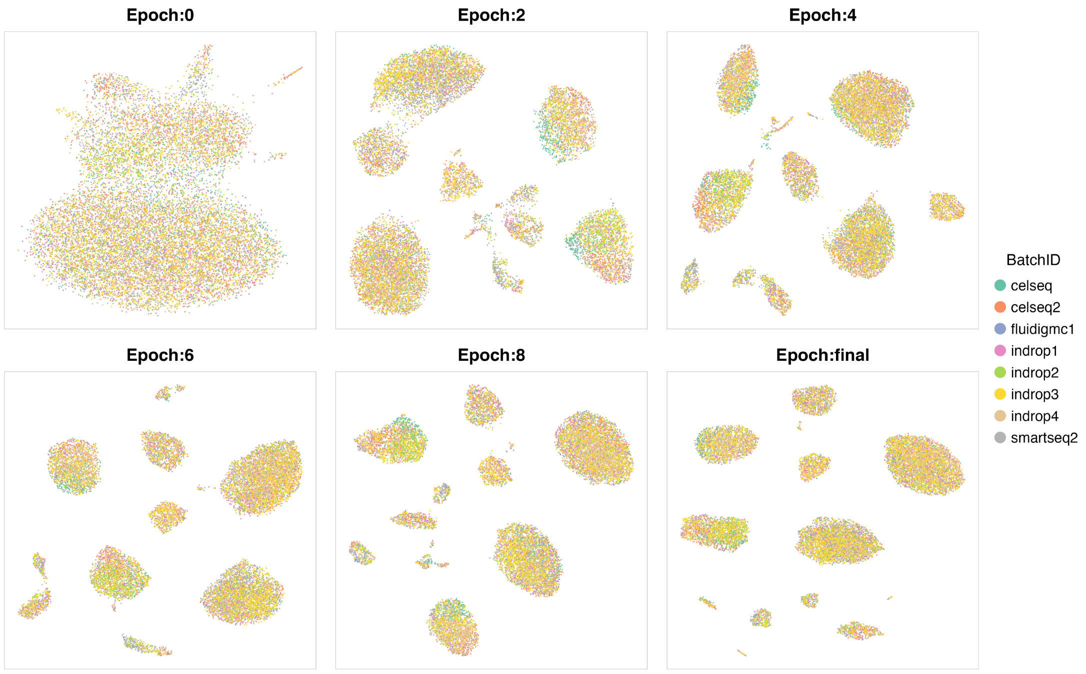

Figure S6. UMAP plots for the pancreas dataset showing that scDML removes batch effect gradually over iterations.

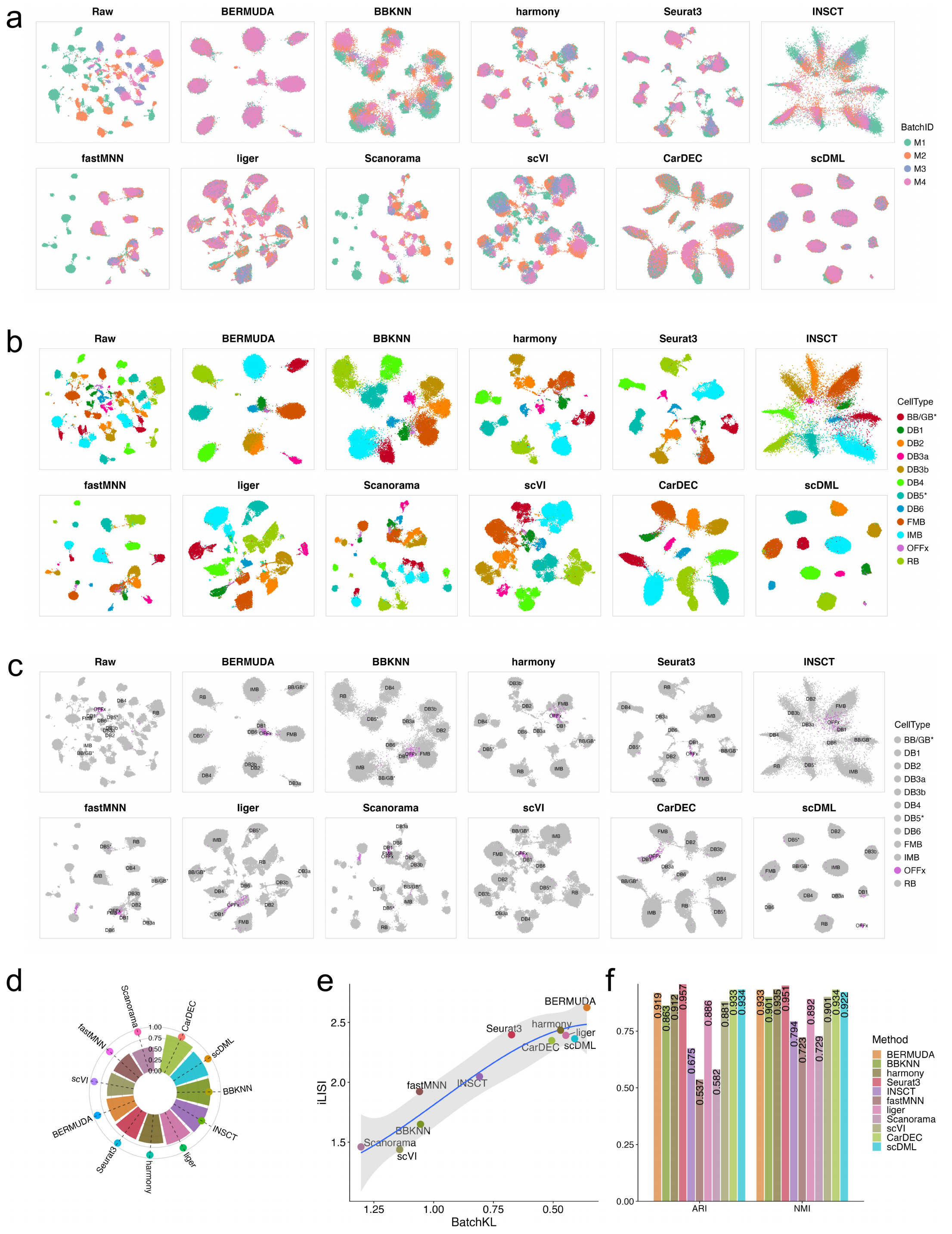

Figure S7. scDML removes batch effects and keeps the biological difference in the macaque retina dataset.

UMAP embedding from different methods, and the points are colored by batch (a) and by cluster label (b).

(c) UMAP embedding computed from compared methods, and the highlighted cell type *OFFx* is the cell type with the fewest cells.

(d) Bar plot shows the value of ASW_celltype and ASW_batch, in which the bar height denotes the value of ASW_celltype and the point height denotes the value of ASW_batch. Higher ASW_celltype and lower ASW_batch means better performance.

(e) Scatter plot shows the value of BatchKL (x-axis) and iLISI (y-axis). Point closer to the upper right means better performance. The error band means confidence interval of 0.95 level around smooth using B-spline smoothing function with degree equal to 3.
(f) Bar plot shows the value of ARI and NMI for different methods. Higher bar means better performance.

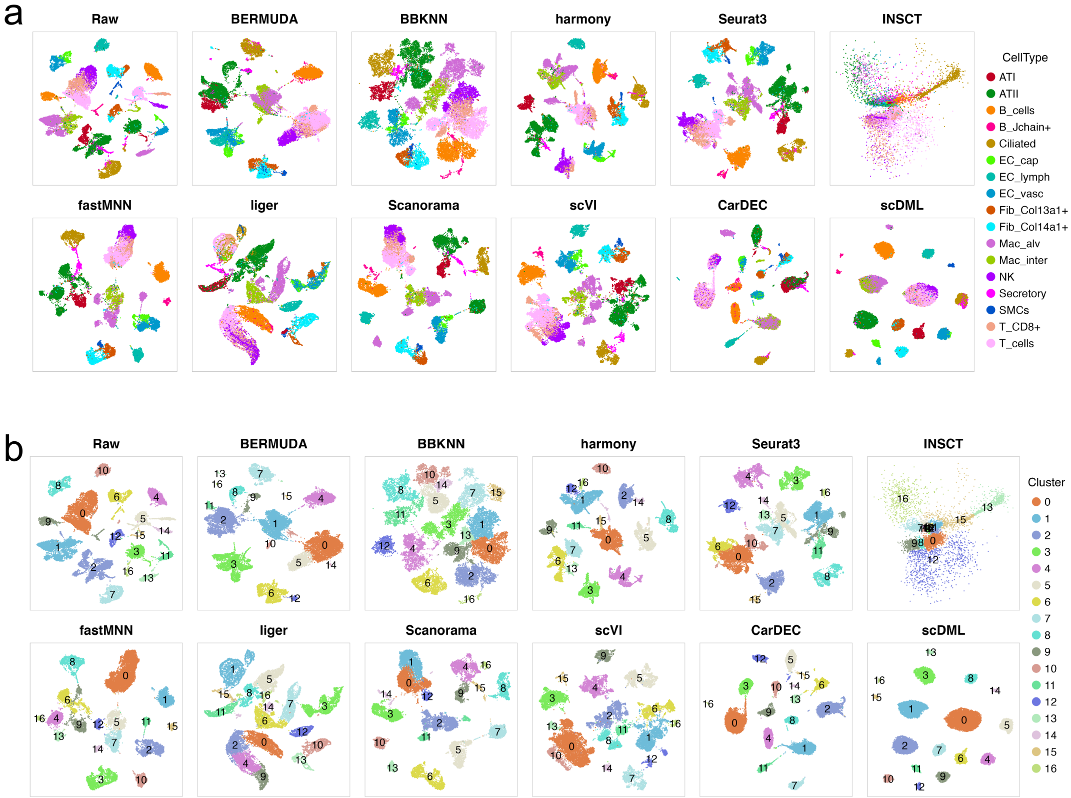

Figure S8. scDML enables cross-species integration for the human and mouse lung dataset.
UMAP embedding from different methods, and the points are colored by batch (a) and by cluster label (b).

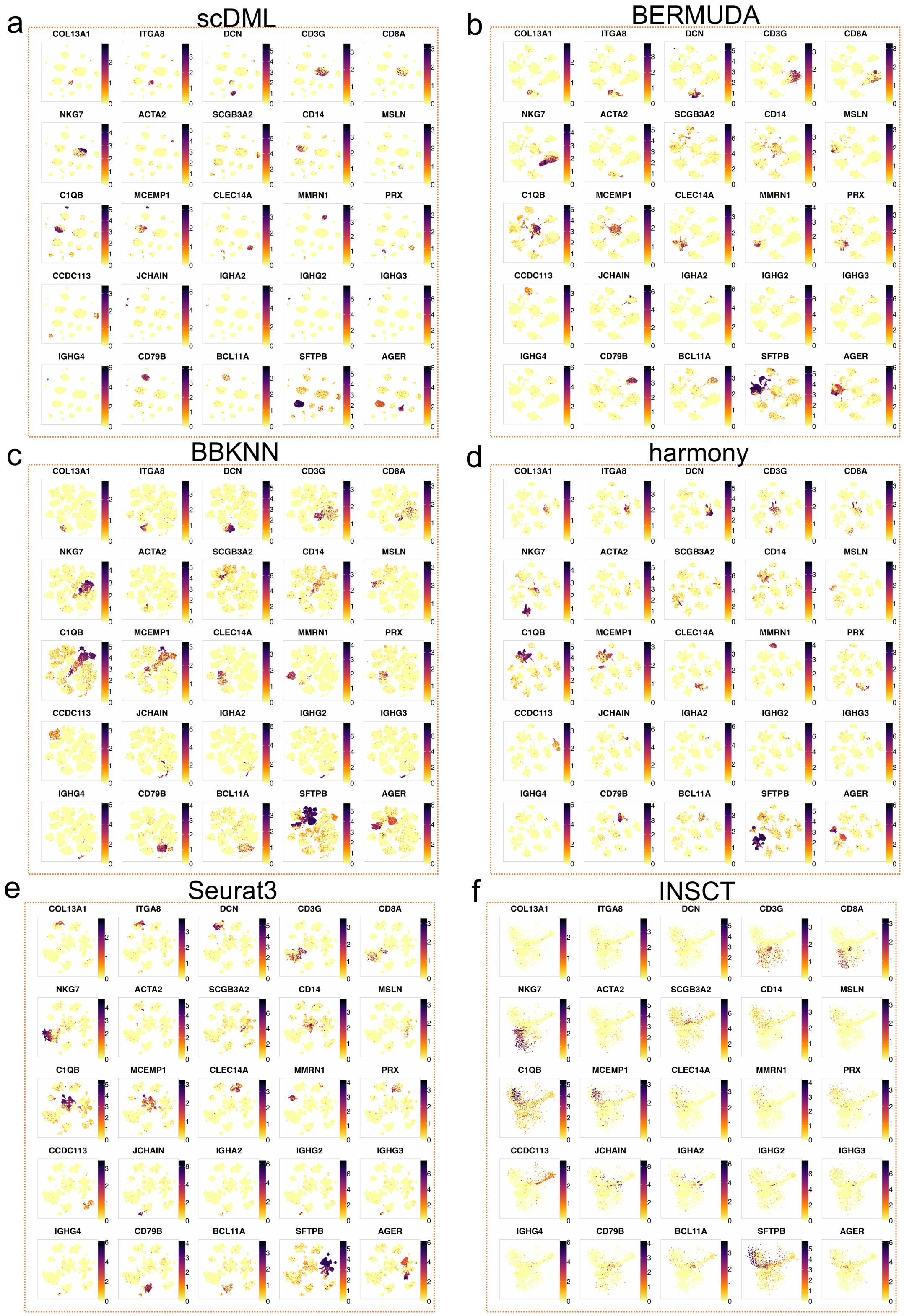

Figure S9. Feature plots show some marker genes of different methods for the lung dataset. (a: scDML, b: BERMUDA, c: BBKNN, d: Harmony, e: Seurat 3, f: INSCT)

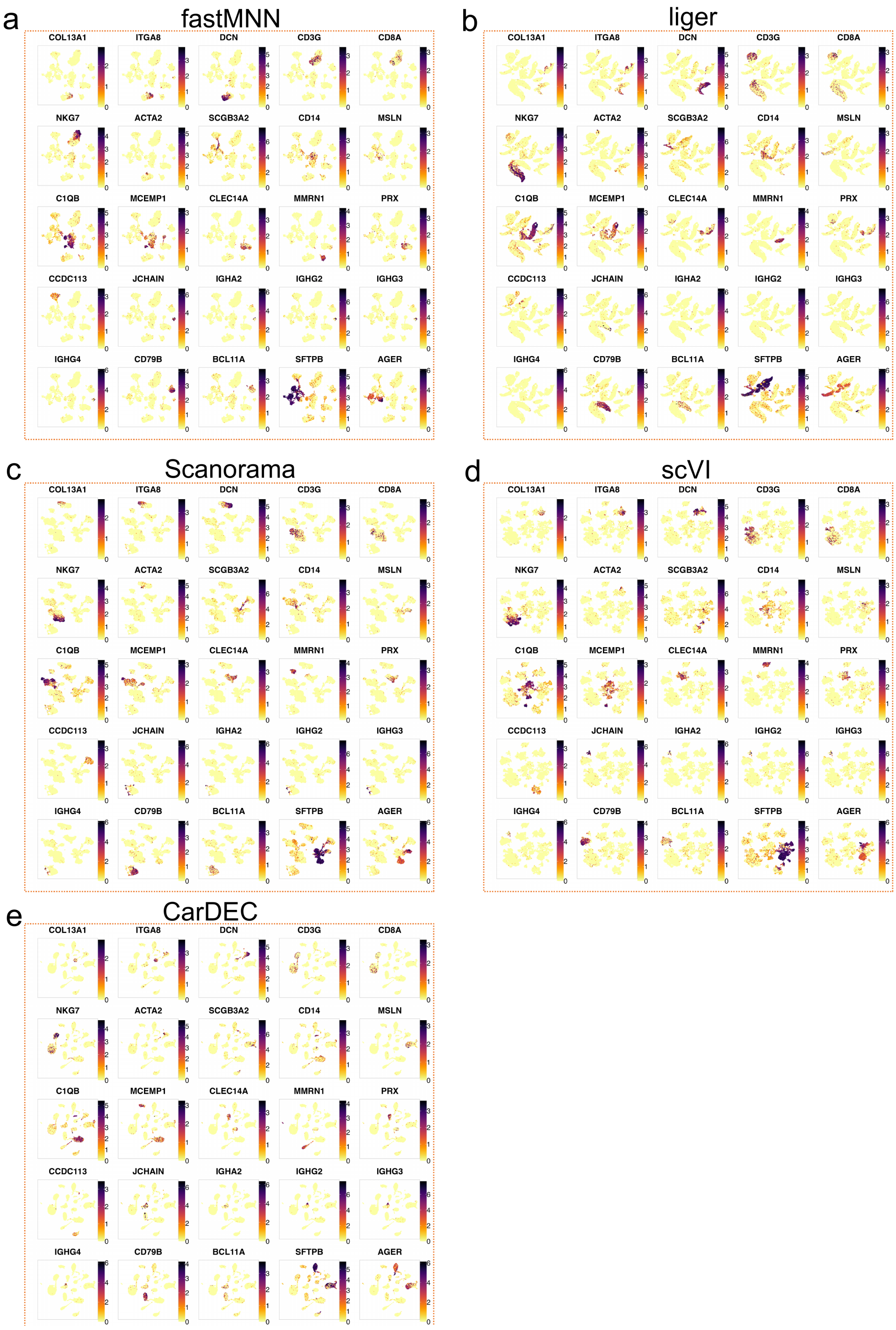

Figure S10. Feature plots show some marker genes of different methods for the lung dataset (a: fastMNN, b: Liger, c: Scanorama, d: scVI, e:CarDEC)

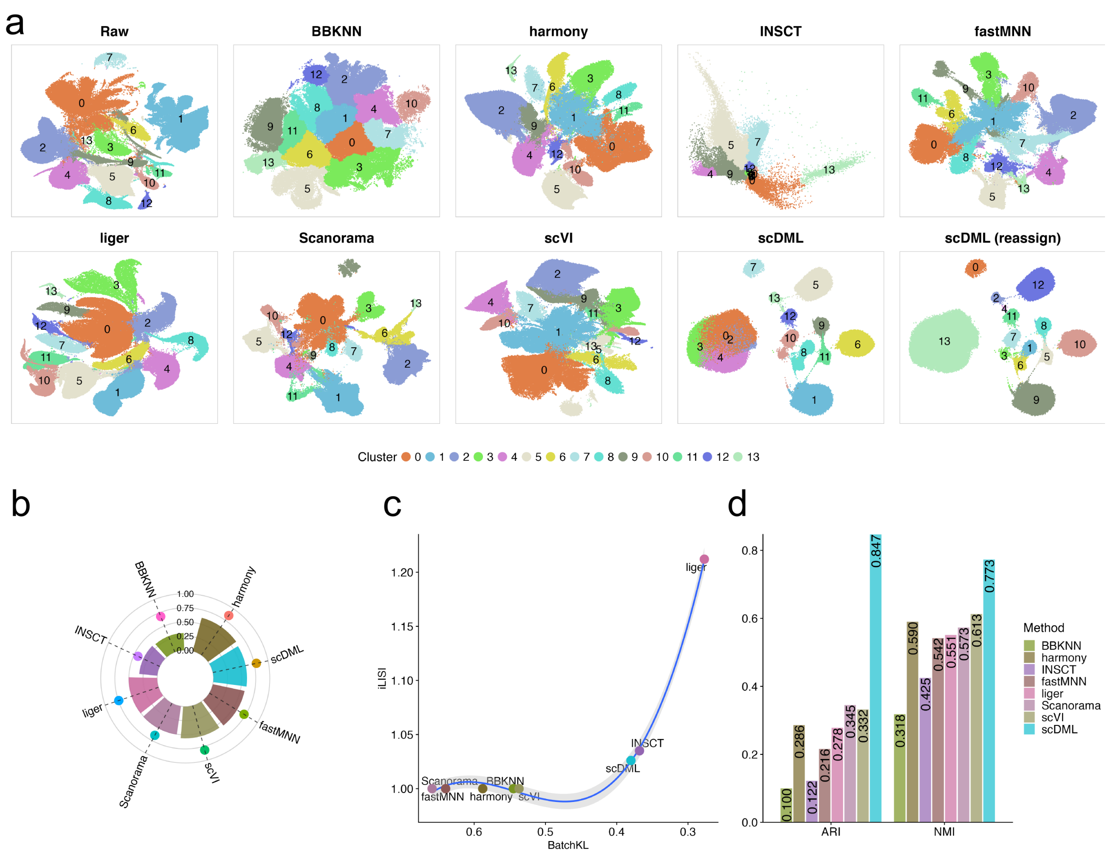

Figure S11. scDML enables integrating large datasets and simultaneously removing batch effect for the mouse brain dataset.

(a) UMAP embedding computed from different methods, in which the points colored by cluster label. The last two sub figures are obtained by using Louvian algorithm based on embedding from scDML and merged clusters based on similarity matrix from scDML, respectively.

(b) Bar plot shows the value of ASW_celltype and ASW_batch, in which the bar height denotes the value of ASW_celltype and the point height denotes the value of ASW_batch. Higher ASW_celltype and lower ASW_batch means better performance.

(c) Scatter plot shows the value of BatchKL (x-axis) and iLISI (y-axis). Point closer to the upper right means better performance. The error band means confidence interval of 0.95 level around smooth using B-spline smoothing function with degree equal to 3.

(d) Bar plot shows the value of ARI and NMI for different methods. Higher bar means better performance.

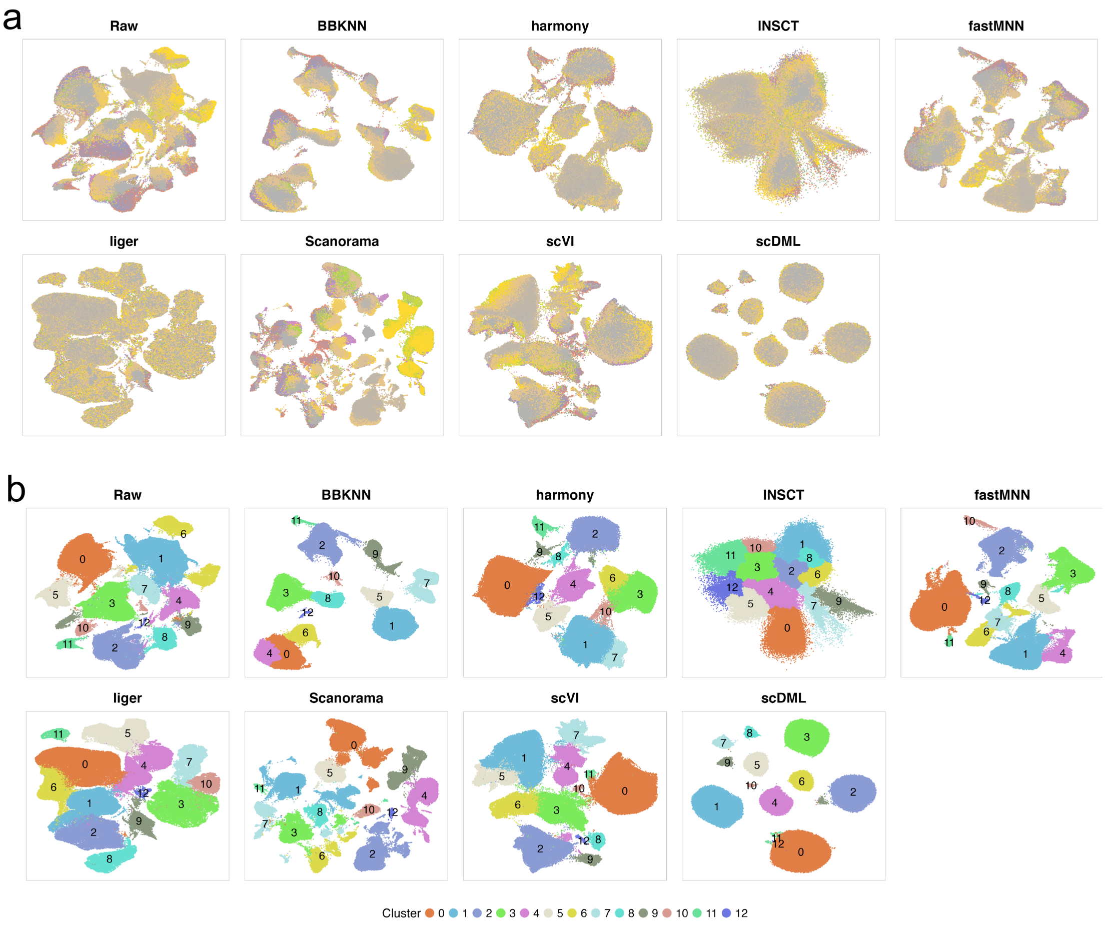

Figure S12. scDML enables integration for the large number of batches on healthy human heart dataset with 140 batches. UMAP embedding from different methods, and the points are colored (a) by batch (140 batches in total) and (b) by cluster label.

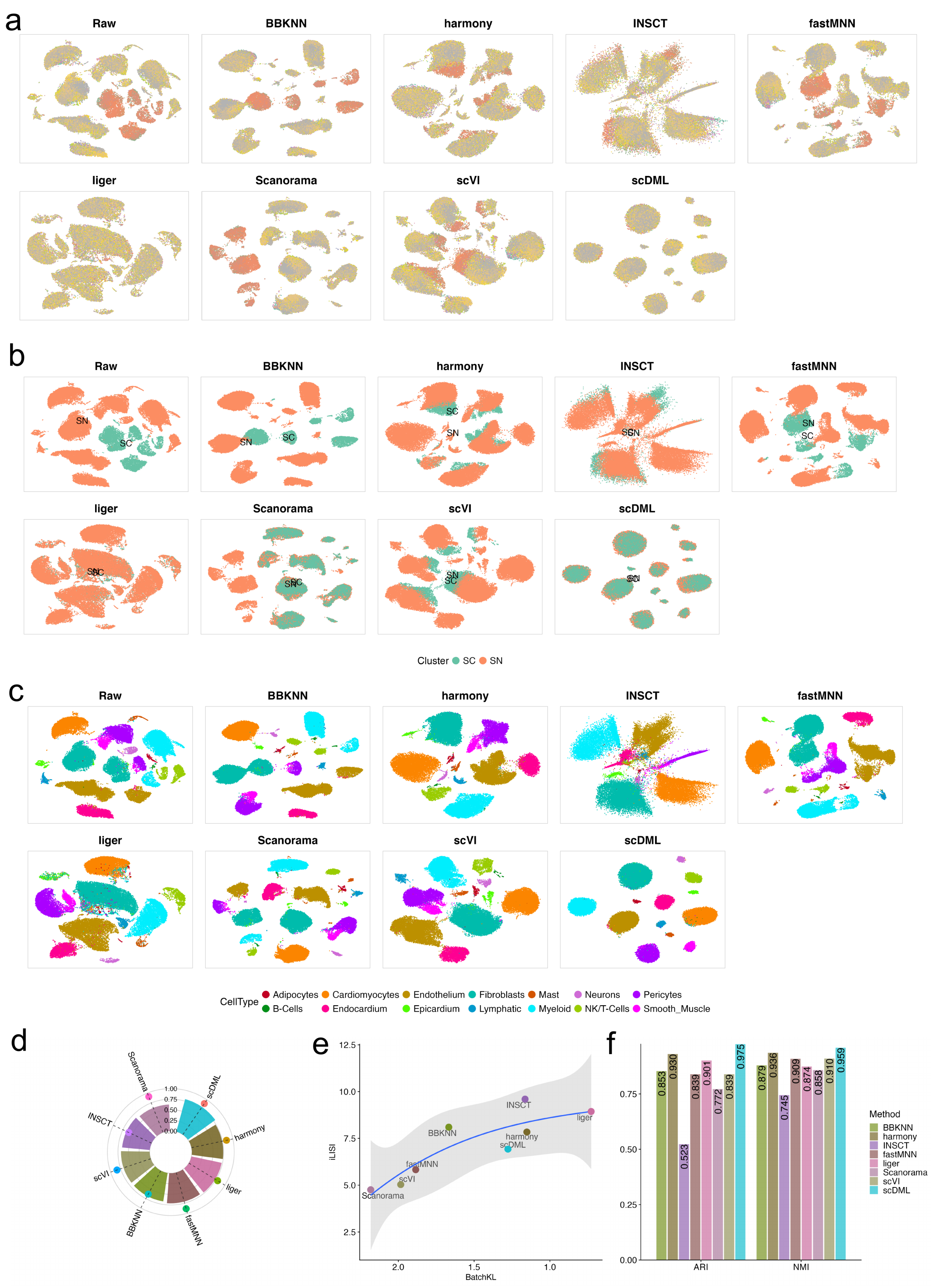

Figure S13. scDML removes batch effects and keeps the biological difference in the failing human heart data with 45 multi-level batches. UMAP embedding computed from compared methods, in which the points are colored (a) by sample (45 samples in total), (b) by technology and (c) by true cell type label.

(d) Bar plot shows the value of ASW_celltype and ASW_batch, in which the bar height denotes the value of ASW_celltype and the point height denotes the value of ASW_batch. Higher ASW_label and lower ASW_batch means better performance.

(e) Scatter plot shows the BatchKL metric (x-axis) and the iLISI metric (y-axis). Point closer to the upper right means better performance. The error band means confidence interval of 0.95 level around smooth using B-spline smoothing function with degree equal to 3.
(f) Bar plot shows the value of ARI and NMI for different methods. Higher bar means better performance.

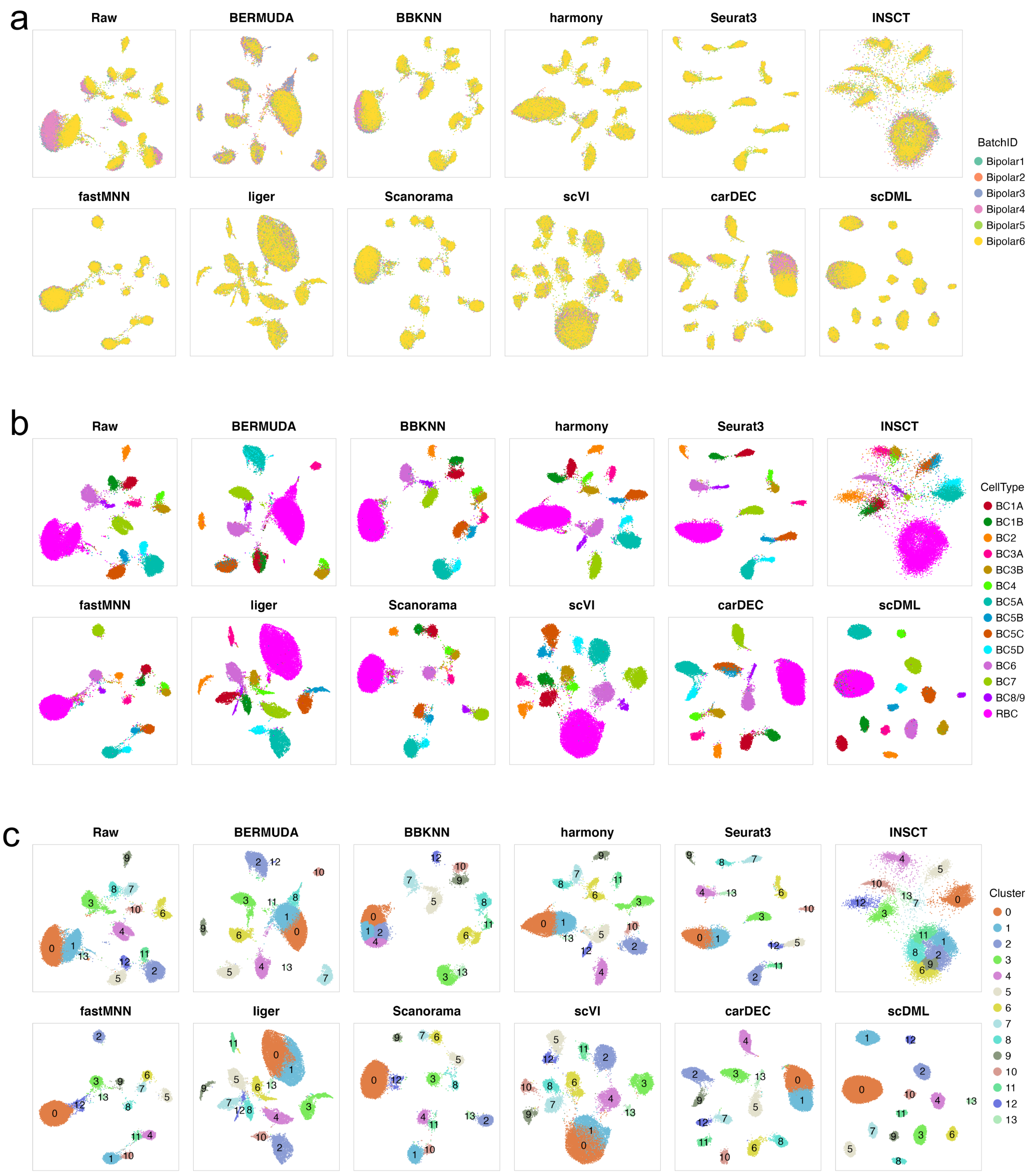

Figure S14. scDML removes batch effects and keeps the biological difference in the mouse retina dataset
UMAP embedding from different methods, and the points are colored by batch (a), by cell type (b) and by cluster label (c).

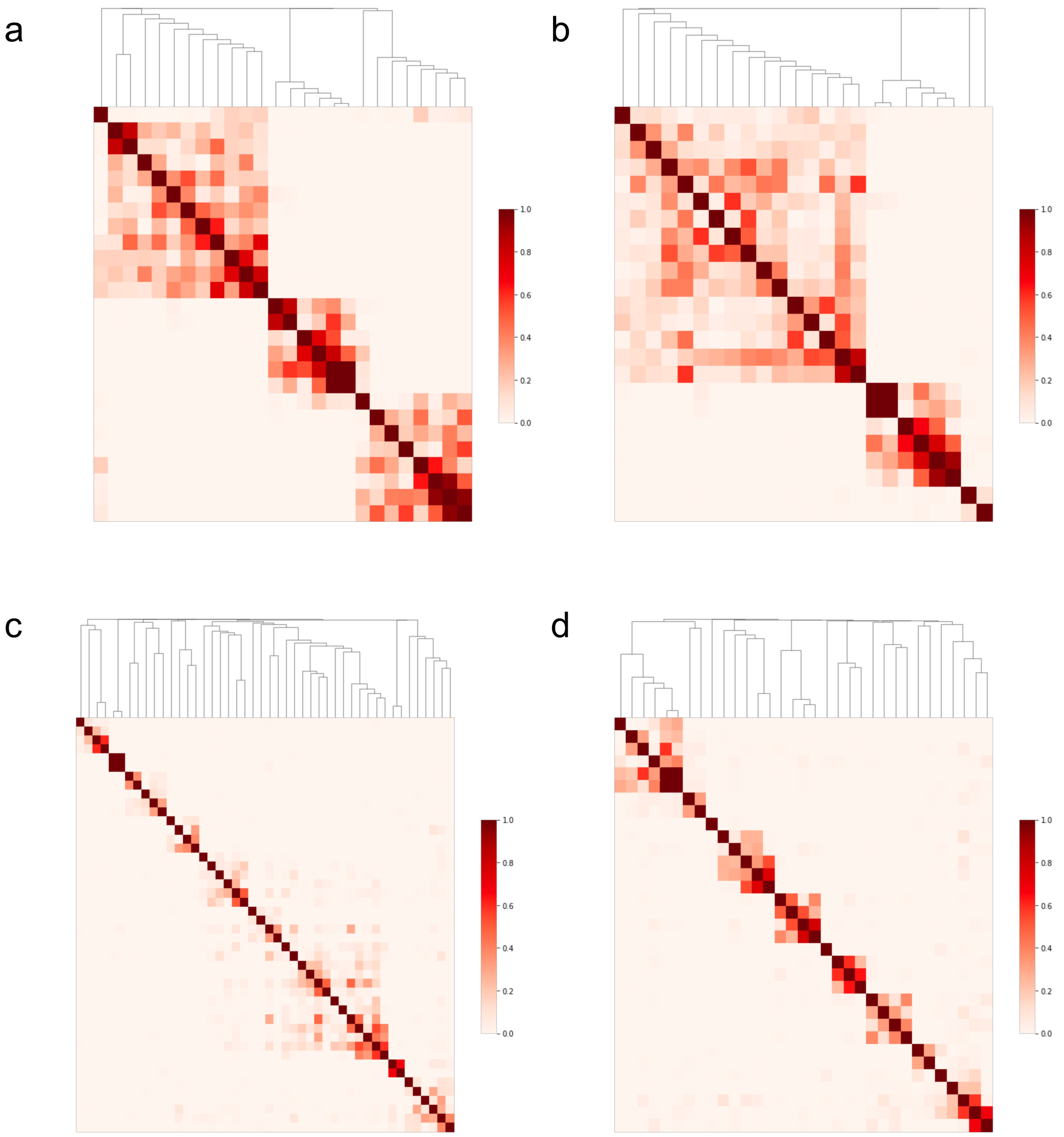

Figure S15. Heatmap shows the hierarchical merging order for datasets bct (a), bct_del (b), human_mouse_lung (c) and macaque retina (d). The suggested number of clusters by Algorithm 3 for all datasets refers to Supplementary Table S5.

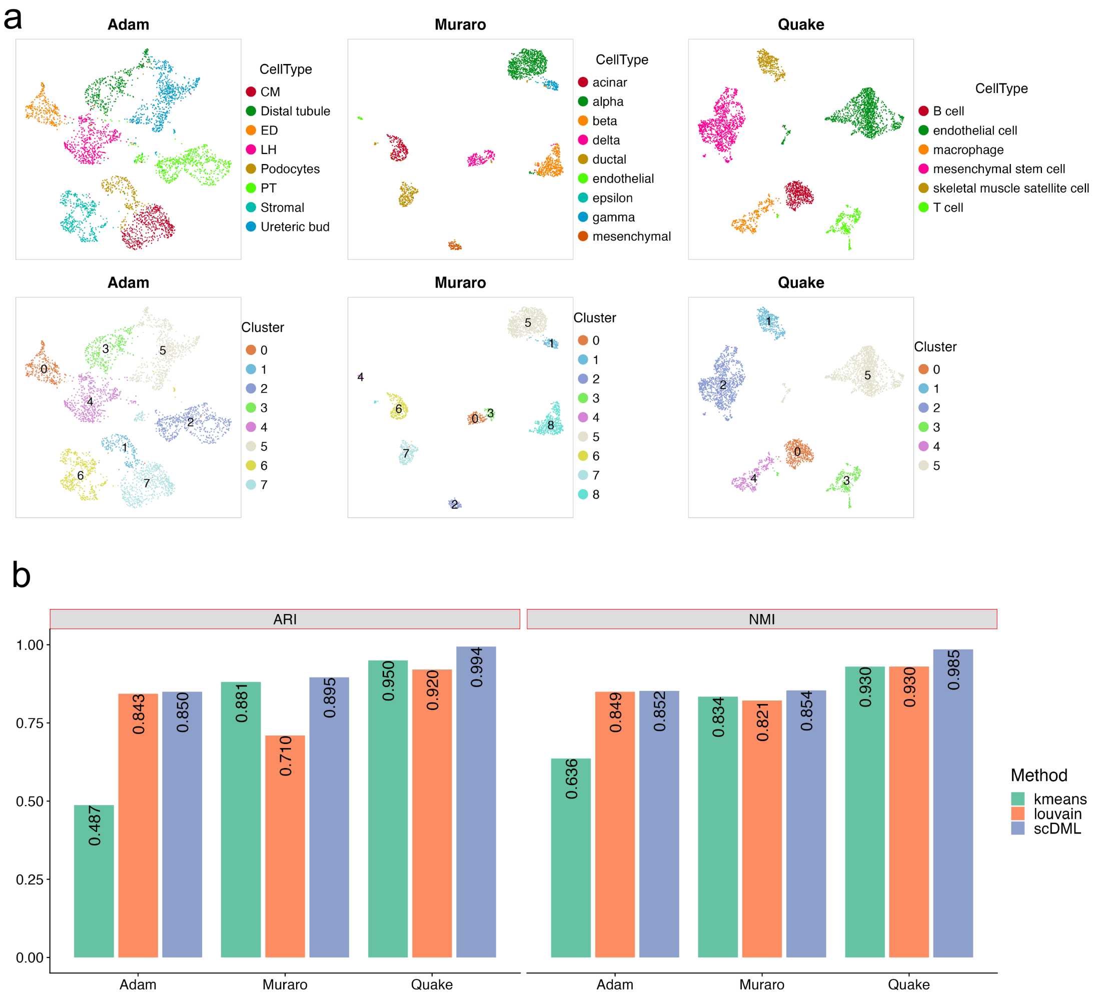

Figure 16. The framework of scDML improves the performance of clustering even there is only a single batch.

(a) UMAP embedding obtained from scDML for 3 different datasets with only a single batch.

(b) Bar plot shows the value of ARI and NMI for 3 different clustering methods (scDML, Kmeans, Louvain) on these three single-batch datasets.

Supplementary References

1. Lun Aaron. A description of the theory behind the fastMNN. *https://marionilab.github.io/FurtherMNN2018/theory/description.html* (2019).
2. Korsunsky, I. *et al.* Fast, sensitive and accurate integration of single-cell data with Harmony. *Nat. Methods* **16**, 1289–1296 (2019).
3. Stuart, T. *et al.* Comprehensive Integration of Single-Cell Data. *Cell* **177**, 1888-1902.e21 (2019).
4. Welch, J. D. *et al.* Single-Cell Multi-omic Integration Compares and Contrasts Features of Brain Cell Identity. *Cell* **177**, 1873-1887.e17 (2019).
5. Simon, L. M., Wang, Y.-Y. & Zhao, Z. Integration of millions of transcriptomes using batch-aware triplet neural networks. *Nat. Mach. Intell.* **3**, 705–715 (2021).
6. Wang, T. *et al.* BERMUDA: a novel deep transfer learning method for single-cell RNA sequencing batch correction reveals hidden high-resolution cellular subtypes. *Genome Biol.* **20**, 165 (2019).
7. Polański, K. *et al.* BBKNN: fast batch alignment of single cell transcriptomes. *Bioinformatics* **36**, 964–965 (2020).
8. Hie, B., Bryson, B. & Berger, B. Efficient integration of heterogeneous single-cell transcriptomes using Scanorama. *Nat. Biotechnol.* **37**, 685–691 (2019).
9. Lopez, R., Regier, J., Cole, M. B., Jordan, M. I. & Yosef, N. Deep generative modeling for single-cell transcriptomics. *Nat. Methods* **15**, 1053–1058 (2018).
10. Lakkis, J. *et al.* A joint deep learning model enables simultaneous batch effect correction, denoising, and clustering in single-cell transcriptomics. *Genome Res.*

31, 1753–1766 (2021).

11. Wolf, F. A., Angerer, P. & Theis, F. J. SCANPY: large-scale single-cell gene expression data analysis. *Genome Biol.* **19**, 15 (2018).
12. Blondel, V., Guillaume, J.-L., Lambiotte, R. & Lefebvre, E. Fast unfolding of communities in large networks. (2008) doi:10.1088/1742-5468/2008/10/P10008.
13. Barkas, N. *et al.* Joint analysis of heterogeneous single-cell RNA-seq dataset collections. *Nat. Methods* **16**, 695–698 (2019).
14. Haghverdi, L., Lun, A. T. L., Morgan, M. D. & Marioni, J. C. Batch effects in single-cell RNA-sequencing data are corrected by matching mutual nearest neighbors. *Nat. Biotechnol.* **36**, 421–427 (2018).
15. Zhang, F., Wu, Y. & Tian, W. A novel approach to remove the batch effect of single-cell data. *Cell Discov.* **5**, 46 (2019).
16. Kaya & Bilge. Deep Metric Learning: A Survey. *Symmetry* **11**, 1066 (2019).
17. Bellet, A., Habrard, A. & Sebban, M. A Survey on Metric Learning for Feature Vectors and Structured Data. Preprint at https://doi.org/10.48550/arXiv.1306.6709 (2014).
