# Supplementary material for: Batch alignment of single-cell transcriptomics data using deep metric learning
Source: Nat Commun. 2023 Feb 21;14:960. doi: 10.1038/s41467-023-36635-5 (PMC9944958; doi:10.1038/s41467-023-36635-5)
Supplement: Supplementary file 3 — Description of Additional Supplementary Files [file 41467_2023_36635_MOESM3_ESM.pdf]

Description of Additional Supplementary Files:

Supplementary Data 1: Datasets analyzed in this paper. All datasets used in our manuscript is also available from : https://doi.org/10.6084/m9.figshare.20499630
