# Supplementary material for: Batch alignment of single-cell transcriptomics data using deep metric learning
Source: Nat Commun. 2023 Feb 21;14:960. doi: 10.1038/s41467-023-36635-5 (PMC9944958; doi:10.1038/s41467-023-36635-5)
Supplement: Supplementary file 5 — Reporting Summary [file 41467_2023_36635_MOESM5_ESM.pdf]

nature portfolio

Corresponding author(s): Xiangjie Li, Jingxiao Zhang

Last updated by author(s): Jan 14, 2023

Reporting Summary

Nature Portfolio wishes to improve the reproducibility of the work that we publish. This form provides structure for consistency and transparency in reporting. For further information on Nature Portfolio policies, see our Editorial Policies and the Editorial Policy Checklist.

Statistics

For all statistical analyses, confirm that the following items are present in the figure legend, table legend, main text, or Methods section.

n/a	Confirmed	
☐	☒	The exact sample size (*n*) for each experimental group/condition, given as a discrete number and unit of measurement
☐	☒	A statement on whether measurements were taken from distinct samples or whether the same sample was measured repeatedly
☒	☐	The statistical test(s) used AND whether they are one- or two-sided<br>*Only common tests should be described solely by name; describe more complex techniques in the Methods section.*
☐	☒	A description of all covariates tested
☐	☒	A description of any assumptions or corrections, such as tests of normality and adjustment for multiple comparisons
☒	☐	A full description of the statistical parameters including central tendency (e.g. means) or other basic estimates (e.g. regression coefficient) AND variation (e.g. standard deviation) or associated estimates of uncertainty (e.g. confidence intervals)
☐	☒	For null hypothesis testing, the test statistic (e.g. *F*, *t*, *r*) with confidence intervals, effect sizes, degrees of freedom and *P* value noted<br>*Give P values as exact values whenever suitable.*
☐	☒	For Bayesian analysis, information on the choice of priors and Markov chain Monte Carlo settings
☐	☒	For hierarchical and complex designs, identification of the appropriate level for tests and full reporting of outcomes
☒	☐	Estimates of effect sizes (e.g. Cohen's *d*, Pearson's *r*), indicating how they were calculated

Our web collection on statistics for biologists contains articles on many of the points above.

Software and code

Policy information about availability of computer code

Data collection	The present study does not involve data collection. We used publicly available datasets to evaluate the performance of our method.

Data analysis	FastMNN(1.10.0),https://bioconductor.org/packages/release/bioc/html/batchelor.html<br>Harmony(0.1.0),https://github.com/immunogenomics/harmony<br>Seurat(4.1.1),https://satijalab.org/seurat/<br>Liger(1.0.0),https://github.com/welch-lab/liger<br>INSCT(0.0.2),https://github.com/lkmklsmn/insct<br>BERMUDA(master branch in github),https://github.com/txWang/BERMUDA (Note: BERMUDA has no version number;)<br>BBKNN(1.5.1),https://github.com/Teichlab/bbknn<br>Scanorama(1.7.2),https://github.com/brianhie/scanorama<br>scVI(0.8.1),https://github.com/scverse/scvi-tools<br>CarDEC(0.8.1),https://github.com/jlakkis/CarDEC<br>scib(1.0.4),https://github.com/theislab/scib<br>scDML(0.0.1),https://github.com/eleozzr/scDML, or https://zenodo.org/record/7535973<br>scanpy (1.7.2) was used for data pre-processing, Louvain's, Leiden's clustering, UMAP visualization.<br>sklearn(v0.22.1) was used for K-mean's clustering.<br>pytorch-metric-learnin(v0.9.95)<br>Rpackage: ggplot2 (version 3.4.0), googleVis(v0.6.12), LISI(v1.0), fmsb(v.0.7.3),dplyr(v1.0.10), tidyr(v.1.2.1),openxlsx(v.4.2.5)

For manuscripts utilizing custom algorithms or software that are central to the research but not yet described in published literature, software must be made available to editors and reviewers. We strongly encourage code deposition in a community repository (e.g. GitHub). See the Nature Portfolio guidelines for submitting code & software for further information.

Data

Policy information about availability of data

All manuscripts must include a data availability statement. This statement should provide the following information, where applicable:

- Accession codes, unique identifiers, or web links for publicly available datasets
- A description of any restrictions on data availability
- For clinical datasets or third party data, please ensure that the statement adheres to our policy

We analyzed multiple published scRNA-seq datasets and two simulated datasets, which are available through the accession numbers reported in the original articles. (1) Simulated datasets: generated by splatter from Luecken et al, which can be accessed by https://figshare.com/articles/dataset/Benchmarking_atlas-level_data_integration_in_single-cell_genomics_-_integration_task_datasets_Immune_and_pancreas_/12420968 (sim1_1_norm.h5ad, sim2_2_norm.h5ad); (2) Mammary epithelial datasets: mammary epithelial cells from three independent studies, and can be downloaded from https://github.com/NBISweden/single-cell_sib_scilifelab/blob/master/datasets/SCE_MammaryEpithelial_x3.rds;
(3) Human pancreas dataset: We used a pre-annotated collection from the tutorial of Seurat (https://satijalab.org/seurat/archive/v3.2/integration.html, standard workflow) with accession codes GSE81076, GSE85241, GSE86469, GSE84133 and E-MTAB-5061; (4) macaque retina datasets: GSE118480; (5) Mouse retina datasets: GSE81904; (6) Mouse brain datasets: GSE116470 and GSE110823, which can be downloaded from http://scanorama.csail.mit.edu/data.tar.gz; (7) human lung and mouse lung dataset: GSE133747; (8) Healthy human heart dataset: https://www.heartcellatlas.org/ (9) failing human heart dataset: GSE183582; (10) Single batch datasets: three datasets analyzed are processed by Chen et al.48 and can be downloaded from https://drive.google.com/drive/folders/1BIZxZNbouPtGf_cyu7vM44G5EcbxECeu (Adam, Muraro and Quake_10X_Limb_Muscle). Details of these datasets are described in Supplementary Table 1 within Supplementary information. All datasets analyzed are available from https://doi.org/10.6084/m9.figshare.20499630.

Human research participants

Policy information about studies involving human research participants and Sex and Gender in Research.

Reporting on sex and gender	No data collection was involved in the present study.
Population characteristics	No data collection was involved in the present study.
Recruitment	No data collection was involved in the present study.
Ethics oversight	No data collection was involved in the present study.

Note that full information on the approval of the study protocol must also be provided in the manuscript.

Field-specific reporting

Please select the one below that is the best fit for your research. If you are not sure, read the appropriate sections before making your selection.

[x] Life sciences [ ] Behavioural & social sciences [ ] Ecological, evolutionary & environmental sciences

For a reference copy of the document with all sections, see nature.com/documents/nr-reporting-summary-flat.pdf

Life sciences study design

All studies must disclose on these points even when the disclosure is negative.

Sample size	No sample size calculation was performed. All data used in this manuscript were taken from public resources and used to demonstrate the functionalities of scDML. We analyzed multiple published scRNA-seq datasets (different tissues, different technologies, different species, and different disease status) and two simulated datasets , and it is sufficient to demonstrate the functionalities of scDML.
Data exclusions	We performed quality control of scRNA-seq data based on the common used and pre-established criteria in this filed.
Replication	All Attempts at replication were successful and can be performed independently.
Randomization	No samples were collected, thus no randomization was performed.
Blinding	All results are based on published data which have been studied in their original publications. Therefore, blinding from investigators is not possible when we reanalyzed the data. Group allocation information was never provided to the computational algorithms.

Reporting for specific materials, systems and methods

We require information from authors about some types of materials, experimental systems and methods used in many studies. Here, indicate whether each material, system or method listed is relevant to your study. If you are not sure if a list item applies to your research, read the appropriate section before selecting a response.

Materials & experimental systems

n/a	Involved in the study
☒	☐ Antibodies
☒	☐ Eukaryotic cell lines
☒	☐ Palaeontology and archaeology
☒	☐ Animals and other organisms
☒	☐ Clinical data
☒	☐ Dual use research of concern

Methods

n/a	Involved in the study
☒	☐ ChIP-seq
☒	☐ Flow cytometry
☒	☐ MRI-based neuroimaging
